# *In vitro* and *in vivo* anti-malarial activity of tigecycline, a glycylcycline antibiotic, in combination with chloroquine

**DOI:** 10.1186/1475-2875-13-414

**Published:** 2014-10-21

**Authors:** Rajnish Sahu, Larry A Walker, Babu L Tekwani

**Affiliations:** National Center for Natural Product Research, Research Institute of Pharmaceutical Sciences, School of Pharmacy, University of Mississippi, University, Kragujevac, MS 38677 USA; Division of Pharmacology, Department of BioMolecular Sciences, School of Pharmacy, University of Mississippi, University, Kragujevac, MS 38677 USA

**Keywords:** Tigecycline, Chloroquine, Malaria, *Plasmodium falciparum*, *Plasmodium berghei*, Glycylcycline antibiotics

## Abstract

**Background:**

Several antibiotics have shown promising anti-malarial effects and have been useful for malarial chemotherapy, particularly in combination with standard anti-malarial drugs. Tigecycline, a semi-synthetic derivative of minocycline with a unique and novel mechanism of action, is the first clinically available drug in a new class of glycylcycline antibiotics.

**Methods:**

Tigecycline was tested *in vitro* against chloroquine (CQ)-sensitive (D6) and resistant strains (W2) of *Plasmodium falciparum* alone and in combination with CQ. Tigecycline was also tested *in vivo* in combination with CQ in *Plasmodium berghei*-mouse malaria model for parasitaemia suppression, survival and cure of the malaria infection.

**Results:**

Tigecycline was significantly more active against CQ-resistant (W2) than CQ-susceptible (D6) strain of *P. falciparum*. Tigecycline potentiated the anti-malarial action of CQ against the CQ-resistant strain of *P. falciparum* by more than seven-fold. Further, treatment of mice infected with *P. berghei* with tigecycline (ip) produced significant suppression in parasitaemia development and also prolonged the mean survival time. Treatment with as low as 3.7 mg/kg dose of tigecycline, once daily for four days, produced 77-91% suppression in parasitaemia. *In vivo* treatment with tigecycline in combination with subcurative doses of CQ produced complete cure in *P. berghei*-infected mice.

**Conclusion:**

Results indicate prominent anti-malarial action of tigecycline *in vitro* and *in vivo* in combination with CQ and support further evaluation of tigecycline as a potential combination candidate for treatment of drug-resistant cases of malaria.

## Background

**Figure 1:**
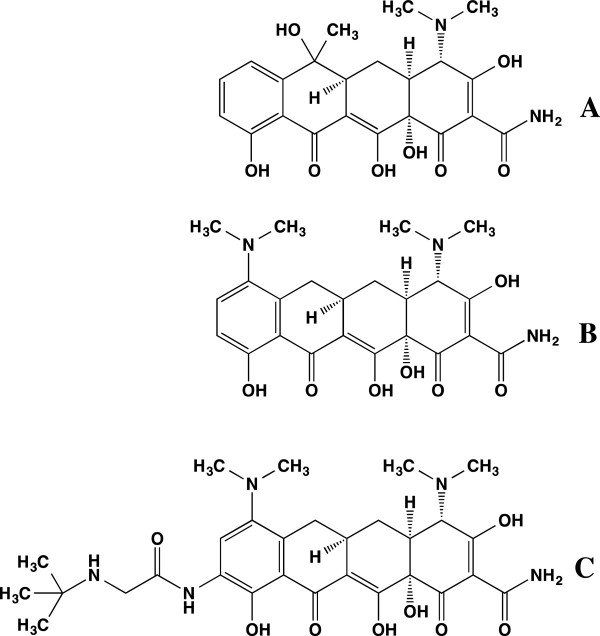
**Structures of (A) tetracycline, (B) minocycline, (C) tigecycline.**

Malaria continues to be the major global health problem and a leading cause of deaths [1]. According to recent WHO estimates, in 2012 there were an estimated 207 million cases of malaria and approximately 627,000 malaria deaths. An estimated 3.4 billion people continue to be at risk of malaria, mostly in Africa and Southeast Asia. Around 80% of malaria cases occur in Africa. Inappropriate treatment or use of chemoprophylaxis, delays in diagnosis or care-seeking, infections with drug-resistant *Plasmodium falciparum* and non-immunity of the individuals exposed to the malaria infection have been identified as important risk factors for malaria-related deaths in individuals living in or travelling to malaria-endemic countries [2]. The spreading of resistance of *P. falciparum* to existing drugs, and recent reports on artemisinin resistance intensify the need for new anti-malarial agents [3–5]. Antibiotics with anti-malarial activity, in combination with traditional anti-malarial drugs, are potentially useful options for drug-resistant cases of malaria [6, 7]. Several antibiotics have shown promising anti-malarial effects and may be useful for malarial chemotherapy in combination with standard anti-malarial drugs [8, 9]. Particularly, tetracycline with quinine or artesunate are considered potential second-line therapy for the treatment of uncomplicated falciparum malaria [10]. A few recent reports have indicated the anti-malarial properties of tigecycline [11–13]. Tigecycline belongs to a new class of glycylcycline tetracycline derivatives with broad-spectrum, anti-infective activities. Glycylcyclines contain glycyl amido substitutions at position 9 (Figure 1). Tigecycline, the 9-t-butylglycylamido derivative of minocycline, is the first marketed compound of this new class of antibiotics [14]. Its increased affinity for the ribosome yields more potent inhibition of prokaryotic protein biosynthesis compared to other tetracyclines [15], and this is thought to be the basis of tigecycline’s improved antibacterial spectrum, including activity against multidrug-resistant species. Tigecycline was found to act faster against *Plasmodium* than any of the other antibiotics tested [13]. It was also tested against clinical isolates of *P. falciparum* from Gabon [11] and the Brazilian Amazon [12]. These studies demonstrate the potential of tetracycline derivatives in the development of improved anti-malarials. The anti-malarial efficacy of tigecycline was evaluated *in vitro* against chloroquine (CQ)-sensitive and CQ-resistant strains of *P. falciparum* and also *in vivo* in *Plasmodium berghei*-mouse model.

## Methods

### *In vitro*anti-malarial parasite lactate dehydrogenase assay

Anti-malarial activity was determined *in vitro* on CQ-sensitive (D6, Sierra Leone) and -resistant (W2, Indochina) strains of *P. falciparum*. The 96-well microplate assay is based on evaluation of the effect of the compounds on growth of asynchronous cultures of *P. falciparum*, determined by the assay of parasite lactate dehydrogenase (pLDH) activity [16]. The appropriate dilutions of the drugs were prepared in DMSO or RPMI-1640 medium and added to the cultures of *P. falciparum* (2% haematocrit, 2% parasitaemia) set up in clear, flat-bottomed, 96-well plates. The plates were placed into the humidified chamber and flushed with a gas mixture of 90% N_2_, 5% CO_2_ and 5% O_2_. The cultures were incubated at 37°C for 48–120 hours. Growth of the parasite in each well was determined by pLDH assay using Malstat® reagent as described earlier [17]. The anti-malarial antibiotics, which are known to act through inhibition apicoplast’s protein biosynthesis [18, 19], induce delayed death of the malaria parasite [20]. The *in vitro* anti-malarial activity of tigecycline and other related antibiotics was also determined at intervals over an extended time period (120 hours) of exposure. The medium and red blood cell (RBC) controls were also set up in each plate. The standard anti-malarial agents, CQ and artemisinin, were used as the positive controls, while DMSO was tested as the negative control. The anti-malarial efficacy (expressed as IC_50_ and IC_90_ values) was determined by the dose response analysis with XLfit©.

### *In vitro*drug combination anti-malarial assay

The potential interactions of tigecycline and CQ were assessed by a ‘chequerboard’ assay design. Serial dilutions of the drugs were prepared in a 96-well plate and tested on non-synchronous parasite cultures. Combination effects were then measured using the pLDH assay for 96 hours as described above [17]. The IC_50_s were calculated for each drug alone and with criss-cross dilutions of the partner drug in both D6 and W2 *P. falciparum* strains.

### *In vivo*anti-malarial assay

The *in vivo* anti-malarial activity of tigecycline alone and combination with CQ was determined by a combined four days’ treatment suppressive-curative-survival assay described earlier [21] in mice infected with *P. berghei* (NK65). The protocol for *in vivo* anti-malarial evaluation has been approved by the University of Mississippi Institutional Animal Care and Use Committee (IACUC). Male mice (Swiss Webster strain) from HARLAN with 18–20 g body weight were intraperitoneally inoculated with 4 × 10^7^ parasitized RBCs obtained from a highly infected donor mouse. Mice were divided into different groups with five mice in each group. Tigecycline was prepared in vehicle (DMSO: Tween-80: PEG-400: water, added sequentially with ratio of 10:0.5: 40:49.5) and CQ was prepared in sterile 0.9% saline. Mice were treated once daily for four days with different doses of CQ (po through oral gavage) and/or tigecycline (ip) about four hours after the infection. Tigecycline was also tested in divided doses, each dose was administered about six hours apart. For combination treatment studies, the mice were first treated with CQ (po) followed by tigecycline (ip) after about two hours. The test drugs were administered to the mice once a day for four consecutive days (days 0 to 3). A control group was treated with an equal volume of vehicle. The mice were closely observed after every dose for any signs of toxicity. The body weights were recorded once daily. Blood smears were prepared on different days starting at five days’ post-infection (until day 28 post-infection) by tail snip, stained with Giemsa and observed under a microscope for determination of parasitaemia. Mice without parasitaemia until day 28 post-infection were considered as cured. Also, suppression in development of parasitaemia was computed by comparing the parasitaemia in control vehicle-treated group and groups treated with test drug or combinations. The mean survival time was also computed for control and treated groups. The results are presented as parasitaemia suppression of day 5 post-treatment, mean survival time of mice in each group, cure and survival graphs computed by Prism 6.0.

## Results

### *In vitro*anti-malarial activity of tigecycline and other related tetracycline antibiotics

The anti-malarial efficacy of tigecycline and other related antibiotics, namely tetracycline and minocycline, was evaluated *in vitro* against CQ-susceptible (Indochina D6) and CQ-resistant (Sierra Leone W2) strains of *P. falciparum* (Table 1). The parent antibiotic tetracycline did not show any anti-malarial activity against D6 and W2 *P. falciparum* strains up to 100 mM concentration, while minocycline and tigecycline both showed time-dependent activity. The inhibition of *P. falciparum* growth by minocycline was faster compared to that by tigecycline. Considering the trend on anti-malarial efficacy, both minocycline and tigecycline were more active against CQ-resistant W2 strain compared to that against CQ-sensitive D6 strain. However, the difference in activity against D6 and W2 strains was much more prominent in the case of tigecycline compared to that by minocycline. Tigecycline exhibited delayed *P. falciparum* growth inhibition phenotype with lowest IC_50_ value at 120 hours, while the trend of inhibition of *P. falciparum* growth with minocycline and CQ was similar with lowest IC_50_ values at 72 hours, which remained constant until 120 hours.

**Table 1:** **Activity of tigecycline and tetracycline antibiotics against chloroquine-susceptible and -resistant strains of** ***Plasmodium falciparum***

Time (hours)	TigecyclineIC _50_(μM)		TetracyclineIC _50_(μM)		MinocyclineIC _50_(μM)		ChloroquineIC _50_(nM)	
	D6	W2	D6	W2	D6	W2	D6	W2
**48**	>100	79.7 ± 20.1	>100	>100	56.7 ± 5.7	40.5 ± 8.58	208.3 ± 10.4	466.0 ± 31.2*
**72**	49.7 ± 1.5	23.3 ± 2.1*	>100	>100	29.9 ± 0.6	25.9 ± 3.08	79.5 ± 0.5	419.0 ± 45.6*
**96**	30.7 ± 2.5	22.0 ± 0.3*	94.3±2.1	89.7 ± 0.6	19.2 ± 2.2	19.1 ± 0.7	79.7 ± 1.5	402.0 ± 2.1*
**120**	23.3 ± 0.6	12.2 ± 3.1*	88.6±1.5	87.6 ± 4.1	27.8 ± 2.4	21.7 ± 1.2	89.7 ± 3.1	397.0 ± 8.1*

Values are mean ± SD of at least three observations.

*-Statistically significant difference (p value <0.05) compared to corresponding activity against D6 strain.

### *In vitro*anti-malarial activity of tigecycline in combination with chloroquine

**Figure 2:**
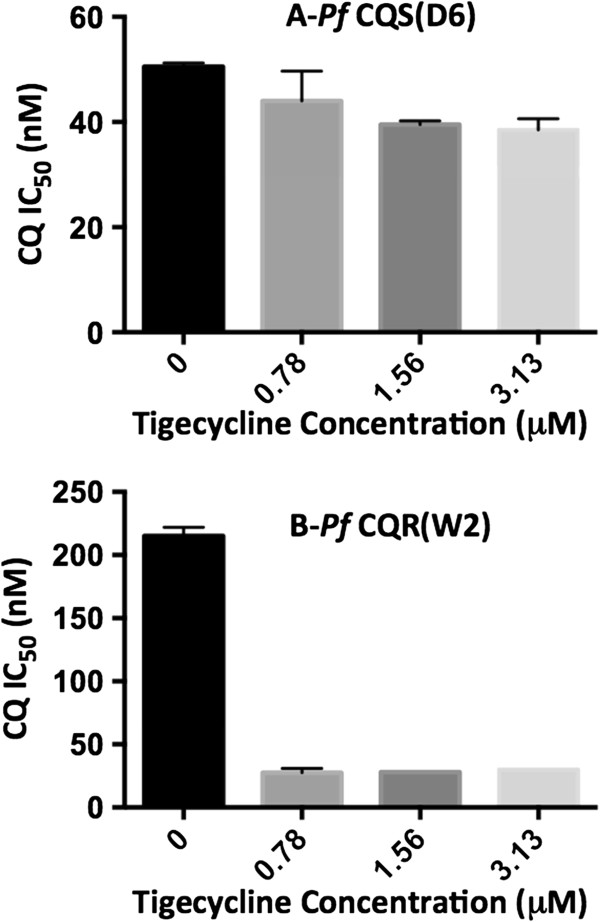
***In vitro*** **activity of chloroquine on CQ-susceptible (D6) and -resistant (W2) strains of** ***Plasmodium falciparum*** **in combination with tigecycline.** Values are mean ± SD of three values.

Further tigecycline was also tested against *P. falciparum* D6 and W2 strains in combination with varying concentrations of CQ (Figure 2). The results obtained with the combinations with higher concentrations of CQ (>50 nM for D6 and >250 nM for W2) and tigecycline (>3.12 mM for both D6 and W2 strains) could not be analysed by the dose response for determination of IC_50_ values due to additive activities at higher doses. The IC_50_ of CQ against both D6 and W2 *P. falciparum* strains in presence of low concentrations (<3.13 mM) of tigecycline were analysed. The IC_50_ for CQ against D6 CQ-S strain did not change significantly in presence 0.78 mM tigecycline (0 *vs* 0.78 p value 0.212). However, the activity of CQ was marginally but significantly improved against D6 CQ-S strain at 1.56 and 3.13 mM tigecycline (0 *vs* 1.56 p value 0.05, 0 *vs* 3.13 p value 0.0388). In contrast, tigecycline produced > seven-fold potentiation of activity of CQ against CQ-R W2 strain of *P. falciparum* (p value <0.0001), even at the lowest concentration tested (0.78 mM) for tigecycline. Tigecycline alone at 0.78-6.25 mM concentrations did not show any noticeable effect on D6 and W2 *P. falciparum* strains.

### *In vivo*anti-malarial activity of tigecycline in *Plasmodium berghei*-mice malaria model

**Figure 3:**
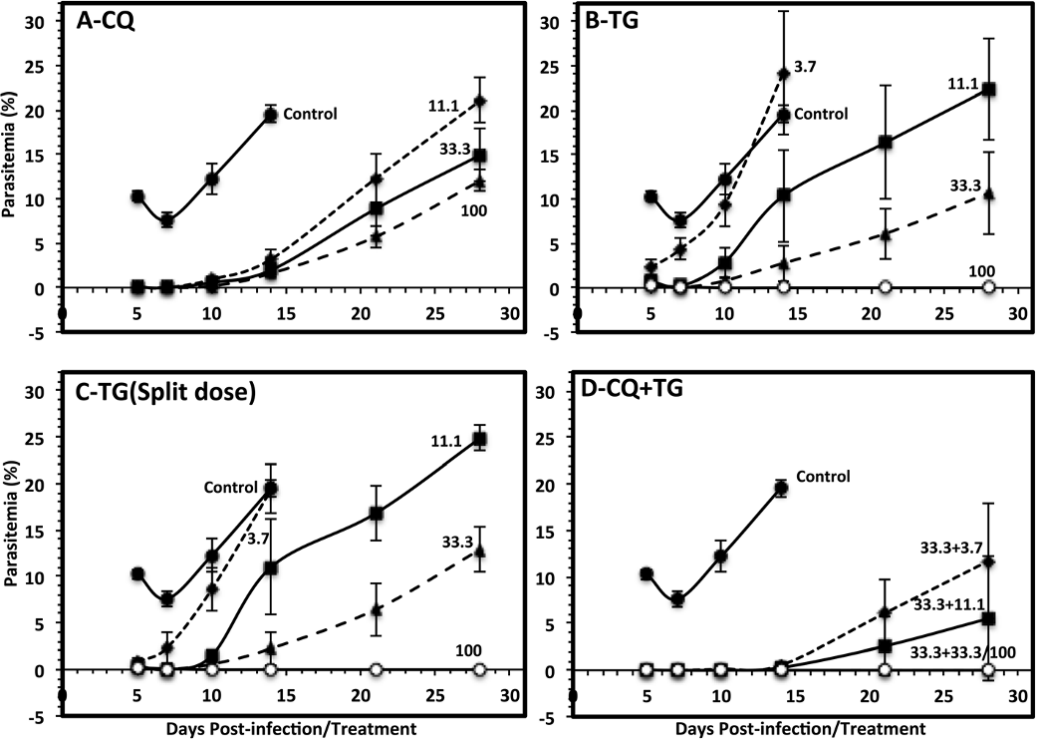
**Progress of parasitaemia in** ***Plasmodium berghei*** **-infected mice treated with [A] chloroquine (CQ); [B] tigecycline (TG) once daily dose; [C] tigecycline (TG) split dose; and, [D] combination of tigecycline (TG) and chloroquine (CQ).** Each point represent value mean ± SD of five mice or the number of mice remaining live at that point of time. The numbers shown along each graph show the daily dose of the specified drug. The mice were treated daily with dose of the drug indicated, for four days staring from day 0 (within 1–2 hours after inoculating the mice with *P. berghei* parasitized erythrocytes. Additional details are described in Methods.

**Figure 4:**
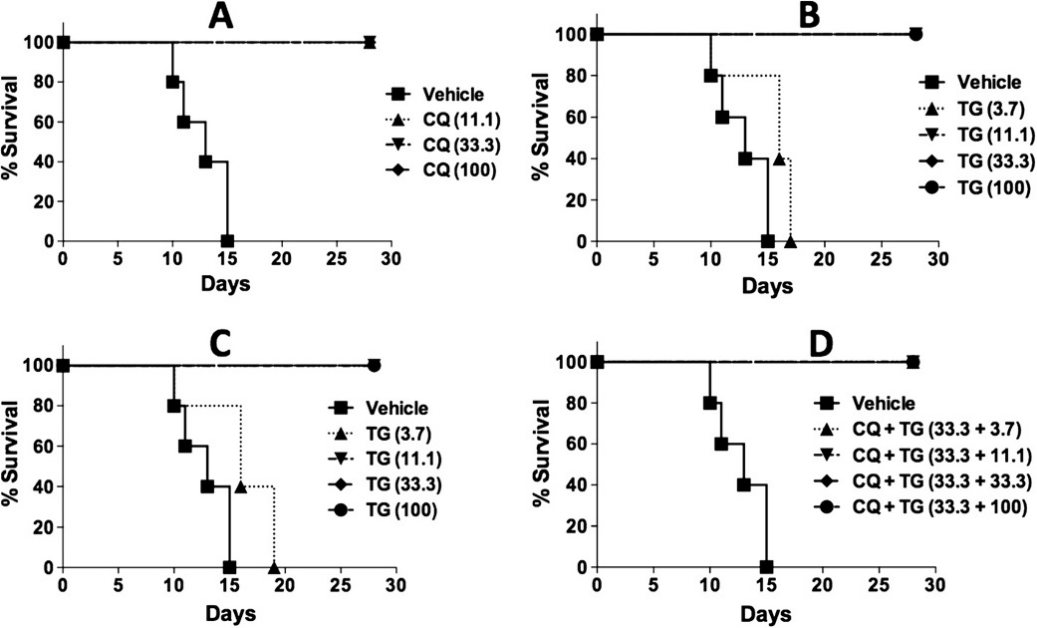
**Survival pattern of** ***Plasmodium berghei*** **-infected mice treated with [A] chloroquine (CQ); [B] tigecycline (TG) once daily dose; [C] tigecycline (TG) split dose; and, [D] combination of tigecycline (TG) and chloroquine (CQ).**

Anti-malarial activity of tigecycline was tested *in vivo* in *P. berghei* mouse malaria model for parasitaemia suppression, survival and cure of the infection (Table 2, Figures 3 and 4). CQ was run as a positive control and also in combination with tigecycline. A control group of mice were treated with the vehicle only. The mice in this group developed significant parasitaemia (8-12%) on day 5 post-infection. The level of parasitaemia marginally declined to (7-9%) on day 7 and increased continuously, resulting in 100% mortality by day 15–20 (MST-12.8 ± 2.3 days). In view of poor bioavailability of tigecycline by the oral route, the drug was tested by the ip route only. Tigecycline was prepared in a specifically formulated vehicle (DMSO: Tween-80: PEG 400: water, added sequentially with ratio of 10:0.5: 40:49.5). This formulation was standardized to achieve maximum solubilization of the drug to ensure its optimum bioavailability *in vivo*. Tigecycline was tested at four dose levels (3.7, 11.1, 33.3 and 100 mg/kg/day) with two different regimens. The first regiment was similar to the control drug CQ, once daily for four days at the doses specified in individual experiments. In another regimen, the daily dose was divided into two equal doses, which were administered six hours apart by the ip route. None of treatment regimens and the dose levels tested for tigecycline produced any noticeable toxic effects in mice. Tigecycline once daily and in divided dose showed similar results regarding suppression of *P. berghei* infection in mice. Interestingly, even the lowest dose of 3.7 mg/kg produced a 77% suppression in parasitaemia on day 5 after the treatment (Table 2 and Figure 3B), and 91% suppression in case of split dose regimen (Table 2 and Figure 3C). However, the low dose of 3.7 mg/kg did not have significant effect on the overall survival or the mean survival time (Table 2 and Figure 4). The level of parasitaemia in mice from these groups recorded on day 14 was also similar to the mice in control vehicle-treated group (Figure 3B and 3C). The control anti-malarial drug CQ produced almost complete suppression in parasitaemia at all the doses (11.1, 33.3 and 100 mg/kg) tested (Table 2). Further, development of parasitaemia in mice from all the CQ-treated groups was significantly delayed (Figure 3A) and the mice in all three CQ-treated group remained alive till 28 days post-infection/treatment (Table 2, Figure 4A) and were euthanized. Higher doses of tigecycline produced >95% suppression in parasitaemia. A rise in the level of parasitaemia was noticed in groups of mice treated with 11.1 and 33.3 mg/kg after ten days post-treatment/infection. The level of parasitaemia in mice treated with 11.1 mg/kg tigecycline reached the level comparable to the control vehicle-treated group by day 28 post-treatment, while the parasitaemia levels in mice treated with 33.3 mg/kg tigecycline remained low, about 50% lower compared to the vehicle treated group at day 28. Interestingly, the higher dose groups, which showed traces of parasitaemia on day 5 and 7 (<0.5%), became free from parasitaemia and remained as such till day 28 (the last day of observation and the experiment). The mice in these groups (100 mg/kg tigecycline single or split dose) were considered cured.

**Table 2:** **Anti-malarial activity of tigecycline in** ***Plasmodium berghei*** **mouse malaria model, alone and in combination with chloroquine**

Treatment	Dose(mg/kg)	Parasitaemia suppression (%) ^1^	Cure ^2^	MST(days) ^3^
Vehicle	-	-	0/5	12.8^±^2.3
CQ	11.1	>99.99	0/5	>28
	33.3	>99.99	0/5	>28
	100	>99.99	0/5	>28
TG	3.7	77.7 ± 9.5	0/5	15.2 ± 2.9
	11.1	93.6 ± 2.7	0/5	>28
	33.3	97.3 ± 1.2	0/5	>28
	100	97.6 ± 0.9	0/5	>28
TG (DD)*	3.7	91.80 ± 3.7	0/5	16.0 ± 3.7
	11.1	96.3 ± 1.7	0/5	>28
	33.3	98.7 ± 0.9	0/5	>28
	100	98.1 ± 0.5	0/5	>28
CQ + TG	33.3 + 3.7	>99.99	1/5	>28
	33.3 + 11.1	>99.99	3/5	>28
	33.3 + 33.3	>99.99	5/5	>28
	33.3 + 100	>99.99	5/5	>28

*(DD)- Divided dose with two split doses six hours apart; ^1^Parasitaemia suppression – Per cent suppression of parasitaemia on day 5 post-treatment compared to vehicle control; >99.99% suppression - no detectable parasitaemia; ^2^Cure number of mice with no detectable parasitaemia until day 28; ^3^MST - mean survival time; MST >28 - all mice in the group survived until day 28 and were euthanized at the end of the study; The data are mean ± SD > of five animals.

Tigecycline was also tested in combination with suboptimal dose of CQ (33.3 mg/kg). Treatment of mice with this dose of CQ, though displaying almost complete suppression of parasitaemia on day 5, was not cured. The parasitaemia in mice from this group rose from day 10 and increased continuously till termination of the experiment on day 28 post-treatment (Figure 3A). Co-treatment of mice with CQ and tigecycline showed a significantly higher anti-malarial effect. At a dose as low as 3.7 mg/kg of tigecycline, 33.3 mg/kg CQ significantly delayed parasitaemia development and produced 20% cure (Figure 3D and Table 2). The co-treatment with 11.1 mg/kg tigecycline in combination with 33.3 mg/kg CQ dose cured 60% of mice (Table 2), and the development of parasitaemia was markedly suppressed in the remaining mice in group (Figure 3D). Treatment with 33.3 and 100 mg/kg tigecycline in combination with 33.3 mg/kg CQ produced 100% cures (Table 2). None of the combination doses showed any noticeable adverse effects in mice.

## Discussion

Use of antibiotics in combination with standard anti-malarial drugs has been an important approach for treatment of drug-resistant, uncomplicated as well as complicated cases of malaria [22]. Antibiotic treatment has been shown to prevent development of malaria infection, and also showed long-term protection in mice against subsequent malaria infections [23]. Prescription of antibiotics in patients with presumed malaria associated febrile illness is high [24]. Doxycycline, a tetracycline antibiotic, is one of the most prescribed, effective and affordable anti-malarial antibiotics [9]. Although it is a slow-acting blood-schizontocide, it is safe and highly effective for treatment of malaria, when used in combination with a fast-acting anti-malarial drug. The anti-malarial use of doxycycline is especially suitable in areas with CQ and multidrug-resistant *P. falciparum* malaria. However, doxycycline confers only partial protection against the sporozoite-induced malaria infections and also use of doxycycline is not recommended for pregnant women and children under eight years of age [9]. Considering the proven records of safety and efficacy, tetracycline antibiotics with appropriate pharmacokinetic and pharmacodynamic profiles should be suitable for inclusion in new combination regimens for treatment of malaria. Attempts to acquire resistance against minocycline in experimental mouse malaria models suggested a slower development of resistance compared to other standard anti-malarial drugs [25]. Recently, in an attempt to optimize anti-malarial efficacy of tetracycline several seven-position modified tetracycline analogues were identified with improved anti-malarial activity *in vitro* against *P. falciparum* and *in vivo* in mouse malaria models [26]. Tigecycline is actually a glycylcycline derivative of minocycline, the first clinically approved for treatment of skin and soft tissue infections, as well as intra-abdominal infections. Recent reports regarding prominent activity of tigecycline against several field isolates of *P. falciparum* [11–13] prompted the evaluation of this antibiotic *in vitro* against CQ-susceptible and -resistant strains of *P. falciparum* in combination with CQ. Tigecycline was found to be significantly more active against the resistant *P. falciparum* strain than the susceptible. Further, low concentrations of tigecycline markedly and selectively sensitized the CQ-resistant (W2) strains to CQ action. It would be interesting to understand the mechanism for more prominent action of tigecycline against CQ-resistant strains and also potentiation of CQ action against resistant strains. Compared to earlier reports on delayed death of the malaria parasites with antibiotics [6, 20, 27], tigecycline seems to show faster anti-malarial action *in vitro*. However, in vitro activity of tetracycline observed in this activity was low compared to that reported earlier [27]. This may be due delayed death of the parasite by tetracycline. Significant inhibition of *P. falciparum* growth was noticed even with 24-hour exposure of *P. falciparum* culture. A proteomic study with *in vitro* culture of *P. falciparum* schizonts treated with doxycycline indicated significant changes in mitochondria and apicoplast proteomes as distinct characteristic for antimalarial action of this antibiotic [28]. Tigecycline inhibits protein synthesis by binding to the 30S ribosomal subunit of bacteria and blocks entry of aminoacyl-tRNA into the A site of the ribosome during prokaryotic translation [15]. Tigecycline inhibits the initial codon recognition step of tRNA accommodation and prevents rescue by the tetracycline-resistance protein TetM [15]. It would interesting to know the mechanism for anti-malarial action of tigecycline. *In vivo* in the *P. berghei* mouse malaria model, treatment with very low doses (3.7 mg/kg) of tigecycline cause significant suppression of parasitaemia, while treatment with high dose of tigecycline (100 mg/kg) resulted in complete cures. Tigecycline also markedly potentiated the anti-malarial action of CQ *in vivo*.

Tigecycline is administered intravenously in the clinic, and its clinical use for treatment of malaria may be limited. The results presented here show prominent anti-malarial action of tigecycline when administered through ip route. This suggests that pharmacokinetic barriers might be overcome with a better understanding of the barrier transport mechanisms. Pharmacokinetic and pharmacodynamic properties of glycylcycline tetracycline antibiotics have not been widely evaluated, although some progress has been during recent clinical evaluations of tigecycline [29]. Further optimization of pharmacokinetic and pharmacodynamic properties of tigecycline through structural modifications or pharmaceutical formulations to improve its bioavailability may afford a faster route for discovery of a new anti-malarial drug.

## Conclusions

Tigecycline, a glycylcycline antibiotic, showed prominent anti-malarial activity *in vitro* against *P. falciparum* and *in vivo* against *P. berghei* infection in mice. The anti-malarial activity of tigecycline was significantly higher against CQ-resistant (W2) than against -susceptible (D6) *P. falciparum* strain. Also, tigecycline selectively potentiated the anti-malarial action of CQ against CQ-resistant *P. falciparum* strain. Treatment of mice infected with *P. berghei* daily for four days with as low as 3.7 mg/kg dose of tigecycline resulted into 77-91% suppression in parasitaemia. Treatment with tigecycline in combination with subcurative dose of CQ resulted into complete cure of malaria in *P. berghei*-infected mice. This is the first report demonstrating *in vivo* anti-malarial activity of a new class of glycylcycline antibiotic. This *in vivo* non-clinical study is a logical extension of recent reports regarding *in vitro* sensitivity of some field and laboratory isolates of *P. falciparum* against tigecycline. Results indicate potential for optimization and application of glycylcycline antibiotics in combination with standard anti-malarial drugs for treatment of drug-resistant malaria infections.

## Abbreviations

CQ: Chloroquine
CQ-R: Chloroquine-resistant
CQ-S: Chloroquine-susceptible
D6: Indochina CQ-susceptible *P. falciparum* strain
MST: Mean survival time
W2: Sierra Leone CQ-resistant *P. falciparum* strain.
